# Taking Care of Business in a Male – Dominated Drug Economy: Income Strategies, Risks, and Opportunities of Women Who Use Drugs

**DOI:** 10.3389/fpsyt.2022.882128

**Published:** 2022-05-17

**Authors:** Torkel Richert

**Affiliations:** Department of Social Work, Malmö University, Malmö, Sweden

**Keywords:** drug use, addiction, women, gender, drug economy, risks, sexuality

## Abstract

**Background:**

Street level drug economies are often described as hierarchical and gender-segregated arenas where men hold high positions and control the supply of drugs, and where women are confined to marginal and low-level positions. Few studies have explored income strategies, risks and opportunities of women who use drugs within drug economies in the Nordic countries.

**Objective:**

The aim of this study was to analyze women's stories about “taking care of business”–making money and securing drugs–in a local drug economy. The study focuses on the women's gender enactments, the strategies they use to achieve success, and the barriers and risks they face in their everyday endeavors.

**Methods:**

This article draws on informal conversations and in-depth qualitative interviews with 27 female drug users in Malmö, Sweden during periods of fieldwork between 2009 and 2012.

**Results:**

The interviewed women had established themselves as entrepreneurs in the local drug economy, working hard for their money. However, only a few held middle or high positions, and all women described encountering gendered obstacles and risks in their efforts to take care of business. The patriarchal and sexualized nature of the drug economy meant special prerequisites for the women's income strategies and gender enactments. Three main income strategies were distinguished in the women's stories: (1) using femininity and sexuality, (2) proving tough and dangerous by using street masculinity, and (3) establishing trust, being professional, and keeping a low profile. These strategies involved different advantages and disadvantages, as well as different types of risk.

**Conclusions:**

The results show that it is possible for women to achieve success in male-dominated drug economies, but that this is associated with major challenges. Gendered social hierarchies, structures and norms seem to influence the women's gender enactments, opportunities and risks. However, factors such as type of drug use, degree of drug dependence and social position, was also decisive for their possibility of taking care of business. This points to the importance of combining a focus on gender with a focus on other determants of power relations and vulnerabilities, when studying the everyday lives of people who use drugs.

## Introduction

This study is about women who use drugs and their ways of “taking care of business”–making money and securing drugs–within a male-dominated drug economy. The study is based on conversations and in-depth interviews with 27 women with a long-term injection use of heroin or amphetamine. At the time of the interviews, the women were all active in the street level drug economy in the city of Malmö, Sweden.

Regular use of drugs such as heroin or amphetamine is expensive to fund. Developing different supply strategies therefore becomes a very important and time-consuming part of the lives of people who use such substances (1, 2). Few people with extensive drug use are able to finance their drug use solely through legal income (3, 4), and they also face several barriers to obtaining and maintaining formal employment, including poverty, homelessness, discrimination and criminalization (5). Although marginalized drug users often move between formal, informal and illegal income-generating activities, most have to establish themselves as entrepreneurs within the illegal drug economy (1, 5). “Taking care of business” often involves a full-time job that requires endurance, knowledge, risk management, skills and resources (6). It involves a lifestyle that differs from the stereotype image of “drug addicts” as passive, powerless and withdrawn (7).

Drug scenes and street level drug economies tend to be heavily male-dominated. These are, to a large extent, arenas where masculine norms and values govern and where men control most of the supply of drugs (8–10). Drug economies are often described as highly gender-segregated and hierarchical labor markets where the majority of women are confined to marginal and low-level positions (11–15). Women in drug economies are often considered less reliable and less able to handle illegal businesses in comparison with their male colleagues–something that Steffensmeier (16) has discussed in terms of an “institutional sexism” in criminal networks.

An important factor affecting the possibility to succeed in street level drug economies is risk management. The risks of dealing, fencing, and of other illicit businesses in street level drug economies have been described as somewhat similar to those of legitimate businesses, including competition, unsecure transactions, liability, and law enforcement involvement (17), but the intensity of these risks are much higher given the illicit nature of the activities (18). Illegal income-generating activities and street-based drug scenes are also to a great extent characterized by structural, symbolic and everyday violence (5, 10). Street level dealers use a range of strategies to reduce risk, including making careful decisions about what to sell, where to sell and whom to sell to, selling in groups, being mobile before and after transactions, dressing neutrally to avoid detection by police, and cultivating a “tough reputation” or displaying “street masculinity” in order to avoid being ripped off or subjected to violence (18, 19). Exposure to risk and risk management may differ between men and women, due to gender norms in society and the patriarchal nature of drug economies. For example, women who use and sell drugs have been shown to be more likely to become victims of violence and theft, whereas men tend to be more vulnerable to police arrest (18).

Research on men and their roles within drug economies has a long history. Research on women in drug economies, on the other hand, has been largely lacking, although the number of studies has increased in recent decades. Much of the existing research tends to either place a strong focus on women's vulnerability, marginalization and powerlessness due to patriarchal structures, or focus on their agency, resourcefulness and empowerment (12, 15).

This study follows the tradition of a few more recent studies on women in drug economies that combine a focus on the structures that pose risks and barriers to women, with a focus on the women's agency and ways of dealing with barriers [see, for example (12, 18–20)].

The overall aim of the study was to analyze women's stories about taking care of business in a local drug economy. I specifically focused on the women's gender enactments, the strategies they used to achieve success, and the barriers and risks they faced in their everyday endeavors.

Theoretically, the study takes its starting point in the concept of “doing gender” (21). According to this perspective, rather than being socialized into stable gender roles, people “do” gender in everyday interactions. How gender is performed depends on the social context, as it takes form in response to a situated discourse on what it means to be a woman or a man. Individuals are constantly assessed on their ability to live up to socially expected gender norms, and are held accountable for their gendered performances (22). In this way, gender enactments are a response to gendered social hierarchies and expectations, but also reproduce and reinforce them (21).

The concept of “doing gender” has subsequently been used, criticized and further developed by, among others, sociologists and criminologists studying women who use and/or sell drugs. For instance, Miller (23) has argued that analyzes based on doing gender has a tendency to replicate gender dichotomies, putting too little focus on the situational and organizational context. A too strong focus on people's adherence to norms about masculinity and femininity may, according to Miller, limit our ability to fully understand inequality and how gendered actions are a response to structural or situational limitations. Viewing gender as situated action on the other hand means recognizing that there are a multitude of masculinities and femininities rather than one static set of gender roles (23). In a later article, Miller and Carbone-Lopez (20) argue for the importance of “moving beyond doing gender” by focusing on how gender and other facets of social inequality intersect, and by complementing the normative aspects of doing gender with a stronger focus on the gendered organizational features of social life. In the article, they combine “doing gender” with an intersectionality approach, also investigating “doing race, class and place.” They further stress the importance of focusing on how gender norms and practices are embedded within, shape and are shaped by local specific social contexts and that gender practices can be dynamic, variable and transformative (20).

In my analysis of the women's stories, I try to combine an actor's perspective–focusing on the women's motives, strategies and actions, with a structural perspective–where the focus is on how the drug economy is stratified and organized and how this affects the women's opportunities and scope for action. Although doing gender and gender inequalities have been the main theoretical focus, I also analyze and discuss how gender intersects with factors such as social situation, drug use and the degree of dependence, when this is relevant on the basis of the women's stories about taking care of business.

## Passive Victims or Successful Entrepreneurs?

During the 1960s and 70s the perspective on drug use gradually changed from one that perceived drug use primarily as a result of psychopathology, to one that described it more in terms of a social and cultural phenomenon (24). In their seminal article, “Taking Care of Business–The Heroin User's Life on the Street,” Preble and Casey (7) challenged the perception of heroin users as weak, apathetic victims entirely controlled by the drug and using the drug as a form of escape. Instead, heroin users were described as resourceful agents making active decisions in their everyday life. However, women were absent from these representations.

Overall, little attention has been paid to women in the illegal drug economy. This is partly due to their status as a minority, but it is also a consequence of women being considered peripheral and not properly belonging to this context. Another explanation is that, historically, research on drug use has been carried out by men, focusing on men (6). The majority of literature on drug use and illegal economies either entirely ignores women or perceives them only through gender lenses, resulting in a focus on family responsibilities, motherhood, promiscuous life styles and psychopathology (8, 14).

Until relatively recently the discourse on women's drug use and the roles women play within the drug economy has been dominated by representations that reflect passivity and powerlessness (14). Studies have often described women as victims lured into drug abuse by men and as dependent on men for their livelihood and supply of drugs (25–27). In representations of men within the drug economy a number of distinct roles appear: the leader, the companion, the master, the servant, as well as the successful dealer and exploited user. By contrast, women are rarely represented in terms other than as subordinate (6). Some studies also tend to describe men's drug use as normative and embedded in masculine cultures of risk-taking and violence, and link women's drug use to deviant behavior and a failure to live up to traditional gender stereotypes and family roles (28).

During the 1990s, a number of studies were published that presented new images of women. Unlike previous studies, these adopted an actor perspective. Women in the drug economy were now also portrayed as successful and proud entrepreneurs, independent of men for their livelihoods, and capable of holding a variety of roles in the drug economy, including leading positions (11, 29, 30). Other studies described how women used “feminine attributes” such as communication skills and family resources to succeed in the drug economy (31, 32), or how women developed strategies to overcome structural barriers (8).

As early as in 1997, Maher (12) drew attention to the two opposing depictions of women in the drug economy: “the first practically denies women any agency and the second over-endows them with it”. In a later meta-analysis of qualitative studies on women in the drug economy, Maher and Hudson (14) summarized the current state of knowledge. They point to both varying and, in some cases, contradictory images of women in the drug economy, but they also identify a number of common and central themes in the reviewed literature. The results show that the drug economy is generally a gender-segregated labor market where women are largely dependent on men to obtain and maintain attractive job opportunities, that women's roles often contain highly sexualized features, that women use a variety of economic and social resources from both the informal and formal sectors, but still primarily hold low and marginal positions within the drug economy (14).

Over the past decade, a number of researchers have attempted to provide a more complex and nuanced representation of women in the drug economy by combining a structural perspective with an actor's perspective and by combining a gender perspective with a focus on factors such as class and ethnicity.

In their study on women methamphetamine users in rural Missouri, USA, Miller and Carbone-Lopez (20) argues for the importance of moving beyond the primarily normative orientation in studying gender, also investigating gendered organizational features of social life including their intersections with other aspects of social inequality such as those of race, class and place. They show how particular narratives of gender–such as supermom, superwoman, and super thin–were used by the predominantly white, rural working class women to position themselves as gendered actors in culturally acceptable ways. These narratives are available and embedded within racialized cultural understandings of white womanhood. The place and social context of the study was also important in understanding the women's gender enactment and opportunities. Miller and Carbone-Lopez found that almost all of the women were involved in the production and/or selling of methamphetamine, and that only a small minority had exchanged sex for drugs. They explain this unusually high level of female participation in market activities by the special features of the local context. The rural community, characterized by small scale and family-oriented business models as well as long-term relationships and close social bonds, worked to the women's advantage (20).

Moloney et al. (18) have investigated gang-involved young men and women engaged in illicit drug sales in San Francisco, California, focusing on their understandings and experiences of risk. They show that the drug economy involves different opportunities and risks for women and men, where discourses about femininities and masculinities and about male and female bodies shape these risks. The female body was seen as increasing vulnerability to victimization but also as providing increased opportunities to conceal drugs. The masculine street seller persona, displaying toughness and “flashiness,” provided the men with respect and power but increased their likelihood of arrest. Another clear difference was that women described a greater concern about the risk of losing their children or not being able to provide for them.

In their study of women street dealers in Oslo, Norway, Grundetjern and Sandberg (15) use a Bourdieu-inspired theoretical framework of “street capital” showing how the women develop particular strategies to succeed in a drug economy that favors men. They emphasize four such strategies: desexualization, violent posture, emotional detachment and service mindedness. These strategies were used as a way of overcoming constraints due to the women's lack of embodied street capital.

In a later study of women in the Oslo drug economy, Grundetjern (33) focused on how the women enact their gendered identities: performing emphasized femininity, performing street masculinity, employing a feminine business model and flexible use of cultural repertoires. She also showed how the women's gendered performances and resources varied with sociodemographic factors such as age, time of entry into the drug economy as well as educational and employment history (33).

The vast majority of studies on women in the drug economy have been conducted in North America and Australia. The two above-mentioned studies from Norway are the only qualitative Nordic studies with a central focus on women's strategies and roles in the drug economy.

In Sweden, two quantitative studies have examined the income strategies of female drug users. The first study showed, among other things, that selling drugs was an income strategy that was equally common as selling sex, and that the women themselves bought most of the drugs they used (34). The second study showed that women with amphetamines as the main drug to a greater degree had legal/formal sources of income (wage labor, pension, social benefits), whereas a larger proportion of heroin users had illegal/informal sources of income (prostitution, dealing, theft) (35). The two studies, however, provide no in-depth insight into what roles or positions the women had in the drug economy, how they perceived their situation or what strategies they used to achieve success–something that the following study aims to explore.

## Materials and Methods

### Recruitment and Interviewing

The study was part of a larger project on drug use patterns, overdose risks and income-generating activities among people who use drugs in Malmö. This article draws on conversations and in-depth qualitative interviews (36) with 27 female drug users recruited at the needle exchange program in Malmö during periods of fieldwork between 2009 and 2012.

The interviewees were recruited through a strategic selection, with the goal of gaining a sample with variation according to age, and type and length of drug use, as well as social situation. This was possible due to the interviewer being well acquainted with the study environment and as a result of continuous fieldwork. Several of the participants were known by the interviewer, as they had participated in a research study about heroin overdoses (37, 38).

The interviews were performed by the author using a thematic interview guide, but much room was left for the free narration of the interviewees. Key themes in the interviews were: the women's background, drug use history and current social situation, experiences of being a woman in a male-dominated context, different ways of obtaining drugs, views on and choices between different income strategies, perceived barriers in making careers and ways to deal with risks.

All interviews except three were carried out face-to-face at the needle exchange program in Malmö or in a nearby park; the remaining three were conducted by telephone after initial contact was made at the needle exchange program. Eight of the women have been followed over a period of several years, by shorter conversations at the needle exchange, telephone contact or multiple face-to-face interviews. The interviews varied in length between 45 and 90 min, and were all recorded and transcribed verbatim.

### Ethics

The study was approved by the Regional Ethical Review Board at Lund University (ref.nr. 2006-346). All interviewees were informed about the study and its aims, as well as their right to end their participation at any time, and all gave their consent to participate in the study. Participants were offered a gift voucher worth 200 SEK (about 22 US$, 20 Euro), for each interview. To ensure the participants' confidentiality, they have been given pseudonyms in this article.

### Analysis

The overall analysis has taken the form of a continuous process where themes, concepts and theoretical ideas have emerged and evolved during the whole project period.

In the analysis of the women's stories, I have used a qualitative textual analysis performed in three steps (36). The first step involved a close reading of all interviews and a summary coding based on the overarching themes in the interview guide. In the second step a more detailed coding was performed, where various patterns–similarities and differences–in relation to the original were identified. Finally, the core themes were interpreted for meaning and suitable illustrative and representative quotes were selected.

### Sample Characteristics

At the time of the interview, the women were between 20 and 63 years, with an average age of 40 years. All of the women were white, only two were born outside of Sweden, and all but one had lived most of their lives in Malmö. All interviewees were cisgender women, and none of the women talked about homo- or bisexual relationships or sexual experiences. Common to all women was that they had been injecting drugs regularly for several years and that they were part of the illegal drug scene in Malmö.

There was a variation in the group in terms of social background, extent and type of drug use, livelihood and current life situation. Fourteen of the women reported heroin as their main drug and 13 stated amphetamine as their main drug, but almost all used several different drugs on a regular bases. Most women reported daily or almost daily use of drugs, but the frequency and quantity also varied greatly over time.

The women had a variety of sources of income. Illegal and informal incomes such as drug sales, thefts, burglaries, fraud and sex sales dominated. But most women also had a formal income, mainly in the form of social benefits or disability pension. Two women had a regular part-time job during the research study period and half of the women had worked at some point in their lives.

The majority of the women had a relatively stable housing situation where they either lived in their own apartment or lived with a partner or friend. However, some of the women described a more unstable situation with temporary housing, and one woman completely lacked accommodation at the time of the interview.

## Results

### Facing Sexism, Stigma and Patriarchal Structures

The women's stories indicated that they were resourceful players in the drug market, but that their opportunities for success were largely limited by prejudices and patriarchal structures. A recurring theme in the women's stories was the obstacles they encountered. These ranged from finding it difficult to secure drugs on credit; to being excluded from participating in burglaries and serious theft; to being offered lower prices for stolen goods while being charged higher prices for drugs; and being forced to provide sexual services in exchange for access to drugs. Several women also described how many men viewed them as easy targets to exploit, cheat or rob.

Within the context of the illegal economy, many lucrative sources of income require good networks and contacts. Those forced to work alone have limited opportunities and suffer great risk of being driven out of business. Maria, a young heroin user, said the following about the opportunity for women to become successful dealers in Malmö.

*It is probably mainly in smaller cities that girls can deal drugs. In Malmö, this is a pretty tough business and there are already so many guys that deal and already have a lot of respect. If a gal enters that business, they will crush her because they have much better gear and better networks. They will make her stop or go bust. And if she takes a man's customers, she will definitely hear of it*.

Petra, who regularly used amphetamine, had succeeded in establishing herself as a low-level drug dealer, but still described similar experiences as Maria. Like Maria, Petra pointed out the difficulty of competing with men as they have better networks. She also said that as a woman, you get “*patronized,” “trash talked”* and “*stigmatized as inferior”* by the men. She explained this with male dealers being the norm, and with the fact that “*the guys feel that we take some of their status away from them*.” According to Petra, the number of female high-level dealers in Malmö can be counted on one hand “*and then I cut my thumb too,”* she added.

Even though the interviewed women had managed to establish themselves as players within the illegal economy, they had to work hard, and encountered much resistance. The most difficult challenge seemed to be getting “*the entrance ticket*” and being able to “*show that you have what it takes.”* Many women described how they were forced to endure sexual harassment and how they had fought hard to break prejudices and win the trust of men in key positions in the drug economy. This is how Johanna, a 27-year-old woman with long-term amphetamine use, described the struggle to be accepted by men in the drug economy.

*I support myself entirely by theft. Mainly copper and scaffolding, and machines. We take everything that pays off, but it can't be too small. Scaffolding is easy, being light metal. Copper is harder work, but it's a good feeling working hard for your money. Girls don't usually get to come out on these jobs, but I've been persistent as hell so they have let me join in the last two years …. I'm absolutely convinced I can go out there and work as well as anybody else. I won't put out for a fix. Not many girls do what I do. It's hard work and then you have to work with men. It's difficult to go up to the guys and say, I don't give it up for a fix, I want to do what you do. It's difficult for them to accept it. I'm accepted now but I've had to work my arse off to get to where I am. But now my word weighs as heavily as anybody else's. It has taken time but it's pretty good now. You have to be persistent, as a girl you have work twice as hard to get half as far as the men, before they understand that you are a hard worker who will dare to go as far as they do*.

The account parallels those of many other women and it clearly depicts the difficulty women encounter when trying to participate and gaining acceptance in male-dominated criminal networks. The women often encounter men that prefer to work with other men and that effectively avoid working with women since they are assumed not to be able to work as hard as men, not to be sufficiently tough, or unwilling to go as far.

Johanna's account not only shows the difficulty of gaining men's trust and respect and thus gaining access to important networks, but also reveals another recurring theme in the women's stories, the sexualized view of women within the drug economy. She described how men expected her to offer sex for drugs (*give it up for a fix*) rather than working with them. She showed a clear pride in being able to work hard for her money under the same terms as the men. Like several of the other women, Johanna also states that using amphetamine made her able to focus and endure long working days and nights.

Josefin told me how she for several years tried to establish herself as a drug dealer, but that she eventually gave it up, because the men she bought drugs from always expected sexual services.

*It's mostly men who deal drugs. As a girl it is mainly guys that you come across, and many guys want... they want more than you want to give, they think they should get something from you, sexually. In this way, you encounter obstacles straight away. That's the experience I have. To be a drug dealer, you have to have contacts that you can buy from cheaply. If you want to buy cheap, many men demand that you have sex with them. If you do not agree to this, you are not allowed to buy*.

For Josefin, the constant sexual harassment and the difficulty in buying drugs at reasonable prices meant that she abandoned her attempts to establish herself as a drug dealer. To fund her drug use, she instead found three regular customers to whom she sold sex. This meant that she did not have to have sex with many different and sometimes unknown men, a steadier and higher income and that she was given more room for negotiation in her sexual contacts. At the same time, she described selling sex as psychologically stressful, leading her to “numb herself” by using more heroin than before.

Another theme in several of the women's stories was the difficulty of combining the role of a mother with a criminal career. Some of the women said that they had opted out of drug dealing or other serious crime because they did not want to risk high penalties and thus lose contact with their children. Lisa, for example, said that she lived a “double life,” where on the one hand she tried to work and live a “normal family life” and on the other hand she took a lot of drugs and was part of the illegal drug economy. She avoided crimes that could result in high penalties, tried to keep a low profile and hide her drug use. She explained her keeping a low profile by not wanting to risk losing her children.

*There are many female drug users like me, who are on the borderline, who work and who take care of themselves. You do not want to lose your children. And it's not as acceptable for a woman to be a drug addict as for a man*.

According to Lisa, being a mother further reinforced the stigma of being a “drug addict,” something that several previous studies have also pointed out (39–41). Other interviewed women said that as mothers, they had poorer career prospects because they were expected to stay at home with children and because they were considered a burden within criminal networks. They depicted a notion, common within the drug economy, that mothers with young children are more likely to “rat” if they are pressured by the police.

It is clear from women's stories that the drug economy in Malmö is primarily governed by men and is based on norms and values that disadvantage women. Two of the interviewed women, however, gave a partially different picture. For example, Sara told me that a growing number of women are dealing drugs, even in middle positions, and that this is gradually becoming more accepted. She compared this with what it was like for her mother and her generation of drug-using women. “*Back then, the choice was primarily between prostitution or being provided for by men.”* According to Sara, there are now more income strategies available for women.

Helen's story also stands out because she said that she did not experience any problems as a woman drug dealer. She believed that the most important thing was to create a good reputation and show suppliers up the chain that you can run the business well.

*The top dealers don't care much about whether you are a man or a woman, they think about who they can benefit most from. I have not noticed that they have a more negative attitude toward women*.

These two stories are exceptions, but they nevertheless indicate that the drug economy is not a uniform or static market and that gender norms and practices may vary within different local contexts or networks. Even in a medium sized city like Malmö (about 325,000 inhabitants), the drug market appears to comprise several different networks, groupings and factions, with partly different cultural norms and dynamics.

### Using Femininity and Sex to Gain Advantages

I did not explicitly aske the women about their sexual orientation. However, based on their rich stories, my view is that the vast majority, if not all, of the participants in the study define themselves as heterosexual women. All described relationships and sexual relations with men. None of the women talked about sexual relationships with other women and the vast majority expressed an overall heteronormative perspective on gender roles, relationships and sexuality.

Although sex and sexuality are used to exert power or to gain advantages in society at large, this seems to be even more evident within the street drug economy that the women are a part of.

Being a woman in a male-dominated and sexualized context could have its advantages. A number of women described how, in different situations, they used their femininity or sexuality to achieve different goals. This involved, for example, playing at sex in order to be offered drugs by men or getting better terms in negotiations, to stalling payments on debts using “female charm,” to playing on an exaggerated or stereotypical femininity to avoid the toughest and most dangerous elements of thefts or burglaries, to achieving higher positions, more status and easier access to drugs by entering relationships with successful criminal men. Julia said that she has used several of these strategies.

*-Is it hard to be a girl in this world?*

“*Yes, but it's probably pretty damn hard to be a guy too, they have a lot to live up to. The advantage of being a girl is that you can use the fact that you're the only girl in a group, you can play stupid, even if I find it difficult. If you're out doing break-ins you can pretend that you can't do this and that. Like, if you stand in a short skirt and high heels and say–so what, I can't do it. You can avoid having to do the hardest or most dangerous things. Then you can use your sexuality in many ways. If you're the only girl you gain a lot of advantages… Then if you're smart you always try to reach the top, as a young girl you look for a guy that's at the top, who has it all. I often look for new contacts to do business with, and then it just happens that we get together*.

In the quote, it is clear how Julia can take on different roles in different situations or contexts. She could display a special kind of femininity in the form of a helpless woman in high heels when needed. However, it is clear from her story that this was not a natural or desirable role for her to play. Later in the interview, she gave examples of when she in other contexts could be powerful, active and tough or how she, in a relationship with her only close female friend, could allow herself to be vulnerable. Most women, like Julia, seem to be able to switch between different roles and femininities, even though the patriarchal and male-dominated context implied certain constrains in most situations.

Later in the interview, Julia emphasized the importance of not becoming too dependent on men. For example, she described how she previously ended up in a very difficult situation when her boyfriend, who was a top dealer, was imprisoned. Because she relied heavily on his income, she had not built up contacts in the illegal economy of her own, or acquired sufficient knowledge to develop her own income strategies.

Like Julia, Paula said that there are opportunities to utilize one's femininity or sexuality to gain advantages. However, she also emphasized that this involves a risky game that requires a skilled balancing act. The risk of getting into dangerous situations or being exposed to sexual abuse is considerable. According to Paula and several other women, it is important to be clear about “*how far you are willing to go”* and to determine when it is time to “*stop the game”* or “*leave the scene.”*

*You often meet drug suppliers that are interested in you sexually. I have been in many situations where I have had an opportunity to gain advantages from being a woman. But you have your own choice. You will always find opportunities to use your sexuality to gain advantages if you want to. That's probably one of the main reasons why women don't get together and form groups and instead work individually in groups of men. You make maximum use of your femininity. Many do so consciously, other subconsciously. You have to use your assets. I think all women use them in some ways, but there are different levels, how far you're prepared to go… Some give it up* [sex] *for a fix. I can use it in the sense that if I'm in debt, I can stall payments, they don't get as pissed off. You're nice to them and charm them. They may think they're getting more than what they're actually getting. Being a young, good-looking girl has its advantages, but it can also be a disadvantage. You easily end up in dangerous situations*.

Both Julia and Paula described themselves as solitary women in male networks. They use this as a strategy to “make the most of their femininity.” At the same time, both said that this means a feeling of loneliness and that they, with one exception, lack close relationships with other women. Although some of the interviewed women had strong friendships and collaborations with other women, the majority primarily spent time with and did business with men.

Malin also said that she has few female friends. She explained that this is due to rivalry between women and competition for men who have status and good access to drugs.

*I only have one best friend, she's a hard-core junkie. Except for her, I have no girlfriends. There is so much rivalry, so much talk about who you are with. It is a competition for the men*.

The patriarchal and male-dominated drug economy seems to imply a competitive situation among many women, something that risks weakening their friendship ties and their position as a group.

In the previous quote by Paula, she emphasized that as a woman, you have your own choice about how you want to utilize your femininity and how far you are willing to go. She made it clear that she was not one of the women who “*give it up [sex] for a fix.”* There was generally a negative view of women who regularly provided sex for drugs. These women generally had a low status, and where considered to contribute to a culture where men generally expect all women to be willing to offer sex in order to get access to drugs. The tendency to emphasis on the women's own choices about exchanging sex for drugs risks putting the blame for the sexualization within the drug economy on the women themselves. This is what Emilia had to say:

*I've never sold sex. It's pretty usual for girls to do that though. There are even girls who do it for a fix. ‘I get my fix and they get what they want’. This makes the situation 10 times harder for the rest of us. All these years, and I can count on my ten fingers those men that have not tried to get sex for drugs. It's a recurring theme. They think you're a walking orifice. Everyone that you buy from tries to exploit you in that way*.

Although several women emphasized use of sexuality as a free choice, it is clear that this was far from a reality for everyone. A hierarchical and sexist drug market where men generally have an advantage in terms of networks, resources, and access to drugs, means limited income options for many women. Most of the women who provide sex to get access to drugs described this as something they were more or less forced to do.

There was however a great variation in sexual practices and attitudes toward use of sex among the women. Some women used sex as a way to make some extra money when interesting opportunities emerged or in periods when other sources of income were limited. For others, the use of sex had become their main income strategy, either by routinely having sex in exchange for drugs, by entering into a long-term sexual relationship with a top dealer or by selling sexual services on the street, at clubs, or to a few regular customers.

Regarding sexuality and sex sales, there were clear differences between the women who primarily used heroin and those who mainly used amphetamines. Most of the women using heroin had engaged in selling sex. Many stated that they really had little interest in sex and that they had few sexual contacts that did not involve payment, something they explained by the suppressive effect of heroin on sex drive or by traumatic sexual experiences. Several of the women stated that they used heroin or benzodiazepines as a way to alleviate the anxiety and pain that the sex sales usually entailed.

In contrast, only a couple of women with amphetamine as the main drug had regularly sold sex. On the other hand, it was more common among women with amphetamines as the main drug to have many temporary sexual contacts. These contacts were sometimes made in order to gain advantages in the drug economy, but several women also said that they made sexual contact with men in the drug economy for pleasure, and that amphetamine facilitated a more lustful, intense and positive sexuality. However, two of women using amphetamine, had a different experience and did not see amphetamine as a sexual drug. One woman described how amphetamine caused her to “*lose the sensation down there*” and another woman said she “*gets numb from the waist down*.” Thus, although amphetamine was generally considered a sexual drug, there were exceptions to this notion.

At the time of the interview, Ella had been using amphetamine for over 20 years and had been active in the drug scene in Malmö for as long. She was one of the women who described the positive effects of amphetamine on her sexuality. With the help of amphetamine she could live out her sexuality and also gain many benefits.

*I use my sexuality on a daily basis. Because it [amphetamine] is a sexual drug. Many girls have pushed their limits and felt raped afterwards, getting hurt. But I have taken care of myself. I acknowledge my sexuality and feel good about it and have a good time, but 99 percent of women in the drug world don't. I can get what I want, I can fuck for diamonds. But I only do it with those I want*.

Ella emphasized that she did not believe that her positive experiences were shared by many other women and stressed the risk of “pushing one's own boundaries” and she also brought up the constant risk of violence and sexual abuse.

Several women described how their ability to successfully make use of their sexuality or femininity, while at the same time avoiding risky situations, was largely related to their current social situation and degree of drug dependence. Severe intoxication, withdrawal symptoms or drug cravings (especially related to heroin) were, according to the women, factors that made it difficult to maintain control over potentially dangerous situations or adhere to self-set boundaries. The women who did not have their own housing, who had limited income opportunities or who had a strong dependence on their main drug, reported few possibilities to select the people they used drugs with or where to sleep, and had more limited room for negotiation in their sexual contacts.

### Using Street Masculinity to Gain Respect

*It's a very male-dominated world. They [the men] can defend themselves better and use a tougher attitude*.

The quote from Hanna above, points to a central aspect of the illegal drug economy–the importance of being able to defend oneself, to be tough and to gain respect. Several previous studies of criminal gangs, street cultures and drug economies show how men have an advantage both in terms of physical size and strength and in the form of symbolic capital of violence, since violence is associated with stereotypical masculinity (9, 42). Street masculinity is a term used to illustrate how the ability to use violence, retaliation and sexual prowess is important to establish a reputation as a “real man” and to maintain a position on the street (43).

Several of the interviewed women described how they used what can be categorized as street masculinity to gain respect. This was especially true for women who held middle or high positions in the drug economy. For some of the women, street masculinity seems to be something they had worked hard to develop or stage, while others described it as a more natural part of their repertoire or identity.

Romina, who mainly financed her heroin use by being a mid-level dealer, stated that using tough jargon and being ready to resort to violence and weapons was a necessity to gain respect and maintain her position. She said that she had always been a tough girl or a “boy girl” and that she had the benefit of growing up among five brothers. At the same time, she said that the extreme toughness she displayed in the drug economy was partly a mask that she had been forced to put on.

*-How do you gain respect as a woman dealing drugs?*

*I've had to work hard for it. But I've always been a tough person. I've had guns and shit. I've been chasing guys with a gun and doing all sorts of crazy stuff out there. I have five brothers so I have learned to be tough. I have never been afraid to fight with guys. I've been through a lot. My life would make a damn good book … There are some girls who are kind and easily deceived, and the men immediately think that they can rob her or beat her. Then there are girls who are tough, who can handle themselves, who set boundaries, who say - if you have money you can get some (drugs), if you don't you can go fuck yourself. You have to be tough to survive. You have to put on a mask, a facade, that's why you can eventually forget who you are, what kind of person you really are*.

The quote summarizes some of the characteristics that are often identified as central to survival in street drug economies: toughness, use of weapons and violence, and the importance of gaining respect. Romina contrasts this with the image of some women who are kind, naive and therefore easy to exploit, rob or beat. For her, it is important to show others in the drug economy that she is not one of the “weak girls.” But as a woman, she has more to prove, she has to be tougher than the men to get the same respect. Romina almost exclusively socialized with men. She described a strong affiliation with the men in the drug economy and identified herself to a greater extent as “*one of the guys”* (23).

Susanna, who supported herself through drug dealing and selling stolen goods, also said she received respect by being cocky and tough and by showing readiness to resort to violence. At the beginning of her career, Susanna often experienced threats, robberies, and sexual harassment. In the end, she realized that she had to set an example in order to be able to continue running her business.

*It has happened that people have come to my home and robbed me. But once there was a guy who didn't get what he wanted, he jumped me, but I had a knife, so I stuck it in his back. A rumor spread around town. I would have done the same today. You should never agree to something you don't want*.

After this incident, Susanna gained a reputation as a potentially dangerous person who did not fear resorting to violence. This led to her experiencing less harassment. Susanna also described how she gradually changed her jargon and her way of dressing to display a tougher appearance. She said she now plays down her femininity, avoids makeup and often dresses in jeans, t-shirt and leather jacket.

Ellinor, who was a high-level dealer, talked about another central code of the street in the drug economy, the importance of not being “a rat.” She believed that there is a notion that women, especially women with children, pose a greater risk in criminal businesses in this regard. According to her, mothers are seen as more likely to rat to the police and as more easily threatened or blackmailed by other dealers because “*they think of their children in the first place*.” Ellinor told me that she chose prison instead of her daughter to show that she was not “a rat.” This meant that she could gain respect and maintain a high position as a dealer even after her prison sentence ended.

*... I had to make that choice and I still stick with it, but I lost my daughter because I kept quiet. They found fingerprints, I could have revealed him and escaped punishment and kept my daughter, but I chose not to tell them whose fingerprints they were. I've been in prison four times without saying anything. It has been difficult to get respect as a female drug dealer. If I had been a guy, everything would have been much easier. But in the end you get respected when they see that you don't talk, and that you don't give in to pressure*.

It is clear that Ellinor, as a woman, had more to prove in relation to her male colleagues. She had to sacrifice more and “go further” in order to get the same respect. She also declared that she, like her male colleagues, generally saw women with children as a greater risk and therefore avoided doing business with them. Few women, according to her, would make the same choices and sacrifices that she has made.

For Romina and Ellinor, as well as for many other women, enacting street masculinity means both pros and cons. The benefits are primarily about winning respect, feeling strong and achieving success in business. The disadvantages mainly concern the risk of “split identity” and loss of relations. Displaying toughness and aggressiveness can be stressful, especially if this is not in line with one's personality or identity. Romina exemplified this when she described how she “put on a mask” and thus risked forgetting who she really was. Other disadvantages that several women addressed were about sacrificing friendships or close relationships, where one extreme example is Ellinor's choice to lose contact with her daughter by not “being a rat.”

Petra described another possible drawback. For her, the display of extreme toughness affected her sense of femininity and the opportunity to start relationships with men.

*We usually have a saying that: the guy must be one level worse than the girl for her to like him. If you are as extreme as me and some other women, it is difficult to find guys. You have to find an extreme guy who is worse than me when it comes to crime and stuff, in order for me to feel feminine. That is what it's all about*.

Common to Romina, Ellinor and Petra was that they held middle or high positions in the drug economy and that they used street masculinity as a main strategy.

### Establishing Trust, Being Professional, and Keeping a Low Profile–An Alternative to Street Masculinity

Far from all the interviewed women were able or willing to display street masculinity. Some stated that they “lacked what it takes,” others believed that there were better strategies for gaining respect and managing business in the drug economy than radiating danger, physical strength and aggressiveness. The women defined a number of such successful strategies, including: to be honest, fair and explicit in business; to deliver what one promised in terms of quality and price; to keep track of your finances and your own drug use; being attentive to customer needs; building trustful relationships with customers and business partners; and keeping a low profile rather than being “cocky” and “brash.”

Monica supported herself primarily through selling stolen goods and dealing amphetamines. She said she had gained a good reputation, respect and good customer relationships by being just and by selling stolen gods and drugs at reasonable prices.

*You have to make people respect you, not that I'm violent or anything, but I have always been straight and fair, you know, with everyone, it doesn't matter who they are and if they want to buy only a gram that's cool too. Many guys, in particular, can get a bit cocky, they're just hard work, saying they won't sell for less than 500 (50€). They get more interesting that way, girls flock around them. But it's only because of the bag of drugs, not because of them, but they suppress all that and think of themselves as top dogs. Most guys are like that, but many of them also fall flat to the ground… If you're fair you manage better, you may get robbed anyway, but most of the time that happens to nasty people, those that are brash and mean to people and cocky just because they walk around with a big bag of drugs. Some people get annoyed when they walk around thinking they're top dogs: it can backfire. People who are treated badly or that are jealous are also more likely to rat on you*.

Having satisfied customers and avoiding annoying people are, according to Monica, important ways to reduce the risk of violent conflicts and of being “exposed to the police.” Given the fact that violent conflicts and police interventions are two of the major threats to activities and incomes in the criminal world, these seem to be important strategies.

Another way of keeping a low profile and avoiding risks used was to dress and act “gender neutral.” This has similarities to what Grundetjern and Sandberg (15) call desexualization and to Deutsch's (44) concept of “undoing gender.” In this case it means neither enacting street masculinity nor using sex or enhanced femininity. According to several women's stories, neutral clothing and a calm attitude reduced the risk of attention, conflict and police intervention compared to street masculinity's flashiness and cocky jargon (compare, 32). To downplay femininity and avoid playing on sex can, according to some women, increase the chance of being respected in the business, while at the same time reducing the risk of sexual harassment and abuse.

Several women said that they were generally less likely than men to be arrested by the police. They explained this with them not being seen as dangerous or criminal to the same extent as their male colleagues. In addition to keeping a low profile, some women described more elaborate strategies to reduce the risk of being arrested by the police. Lisa, who was dealing heroin, told me that she sometimes went down to the red-light district and showed herself to the cops *so “they wouldn't suspect anything else.”* Since sex sales are not criminalized in Sweden (but sex purchases are) it meant less risk to her if the police thought she was selling sex rather than dealing drugs, something that can result in long prison sentences.

Most women emphasized the importance of creating and maintaining good and trustful relationships with other people in the drug economy. All the women were dependent on continuous and secure access to drugs, and most did daily business with others in the form of dealing drugs, selling stolen goods or collaborating on theft or burglary. Drug economies are often depicted as fierce and ruthless arenas characterized by competition and lack of solidarity. Based on the women's stories there is evidence that speaks both for and against this notion. Expressions such as “*you can't trust anyone”* and “*it's about eating or being eaten,”* clearly speak for lack of solidarity and trust. At the same time, several women also gave examples of good collaborations and close relationships.

A few women collaborated with other women, either around sex selling or dealing drugs, some describing this at a kind of “sisterhood.” However, it was far more common among the women to have male business partners. Some women worked with a man who they lived with, others did errands for men higher up in the hierarchy, and two of the women stated that they themselves employed men to do errands for them. Business partners provided access to important contacts and networks, acted as a backup if the business went bad, and provided security reducing the risk of being robbed or exploited.

Drug dealing, in particular, seemed to require good networks and stable relationships. It was important to establish cooperation with one or more top level dealer for a continuous supply of drugs at a good price, and to build a loyal and secure clientele. Gaining a reputation of being reliable at all levels of the distribution chain was described as crucial. When I asked Ylva what she thought were the most important qualifications for gaining a good reputation and becoming a successful dealer, she answered:

*That you have a good supplier. That you do the right thing, and that you always pay your supplier. That you keep a good price and that you always have access to good quality drugs. To keep away from the cops … Being reliable in business is the most important thing, and also being selective with whom you do business with*.

Ylva went on to say that she only sold drugs to a small group of people with whom she had a close relationship, and that she only let people she trusted into her apartment. She refrained from dealing to customers who she perceived as threatening or uncertain, and avoided competing with other dealers about customers. Ylva, like several other women, pointed out that the most important thing was to keep on good terms with their main supplier.

It was common, as Ylva did, to receive drugs on credit which you then sold, repaying the debt with part of the profit. Like all the other women interviewed, Ylva used drugs herself. This can entail a risk in the form of using some of the drugs you plan to sell and thus having difficulty repaying the debt or credit to the main supplier.

One of the most difficult challenges, according to the women who dealt drugs, was not to adjust the consumption to the quantity of drugs in possession. Especially those who both used and sold heroin described it as difficult not to increase their dose and not to “*get high on your own supply.”* This was particularly difficult when the business was bad or during periods with psychological distress or severe cravings or withdrawal symptoms.

Some women said that they managed to control their drug use by setting clear rules for how much they would use, with whom and where. Others said that they conducted their drug sales in the form of a “*professional and regular business,”* with accounting and clear rules and procedures. However, most of the women said that at some point they had lost control over their consumption, which meant that they had to dilute the drugs they sold too much or that they missed payments up the chain. This could lead to a bad reputation and increase the risk of sanctions, something that forced some of the women to periodically abandon drug dealing for other income strategies such as sex sales, fraud or theft.

Several of the women described themselves as professional and successful entrepreneurs and compared their illegal activities with “*regular jobs or businesses.”* According to Lisa, successful drug dealing requires similar qualifications as many other professions where one must be “*ambitious, cooperative, straightforward, honest, strong, and stand up for what you believe in.”* Other important capabilities and norms that the women emphasized were being responsive to customer needs, always delivering what you promise, and practicing an ethical cod of not selling to minors or people not involved in the drug economy. Most women showed great pride in being self-sufficient and working hard for their money.

## Discussion

The interviewed women had all established themselves as entrepreneurs in the local drug economy. They bought most of the drugs they used, and they worked hard for their money. The results differ from those of early studies on women in the drug economy that portray them as highly marginalized or passive victims, dependent on men for their livelihood and access to drugs (25–27, 45).

Similar to a number of more recent studies, the results show that it is possible for women to find their own income strategies and become relatively independent and successful entrepreneurs in illegal drug economies (6, 15, 20). At the same time, only a few of the interviewed women held middle or high positions, and all the women said that they encountered obstacles and challenges in their efforts to take care of business and make careers in the drug economy (compare, 14). Many of these obstacles had to do with being a woman in a male-dominated context.

The street level drug economy in Malmö, like many other illegal economies, appears to be male-dominated, patriarchal and strongly sexualized (9, 14, 15). The women said that they were generally considered less tough and easier to rob, and that they were seen as inferior business partners in comparison to men. Women's limited opportunities in illegal economies have previously been explained by institutionalized sexism (12, 16, 42, 46). This sexism appears primarily in “homosocial reproduction,” “sex typing” and the “task environment of crime” (16). Male criminals prefer to work and do business with other men, meaning that men who occupy leading positions subsequently tend to place other men in significant positions (homosocial reproduction). Male criminals are less prone to work with or do business with women as they are seen to lack the physical and psychological attributes (sex-typing) that are required when working in an instable and violent context (task environment of crime) (16). The women in Malmö clearly experienced institutionalized sexism, and also encountered a sexualized view according to which they were expected to provide sexual services for access to drugs, something that has been described in an earlier study on the Malmö drug scene (34).

The fact that the drug economy was patriarchal and sexualized meant special prerequisites for the women's income strategies and gender enactments. The women's stories must also be understood in the light of the fact that they are cisgender women, all having sexual relationships with men. The overall heteronormative views on gender roles and sexuality held by the women may thus be related to their gender identities and sexuality, as well as to context specific features of the drug economy.

Three main strategies were distinguished in the women's stories: *using femininity and sexuality*, proving tough and dangerous by *using street masculinity*, and a third strategy that involved *establishing trust, being professional, and keeping a low profile*. Some of the women combined different strategies or switched strategy depending on context, while others mainly stuck to one. The three strategies should not be seen as fixed, inseparable or as the only possible ones. Rather, they should be considered as three typical strategies common among the women interviewed.

The three strategies were associated with different types of risks, advantages and disadvantages. Using sexuality and sex enabled relationships with men in high positions in the drug economy, improved access to money and drugs, and made it possible to avoid having to do the most dangerous jobs. Thus, there could be some benefits to being a woman in a male-dominated context. At the same time, using sex could, according to the women, involve increased risks of sexual abuse, less respect and a lower social status. Sexual harassment and abuse appeared to be normalized to some extent–something you can expect to be exposed to as a woman in the drug economy. Using sex as a main strategy also seemed to be associated with greater loneliness–this made it hard to be “one of the guys” and at the same time limited the opportunities for close friendships with other women, due to bad reputation or competition for men. Grundetjern (33) has used the concept of emphasized femininity to show how some female dealers in Oslo's drug economy played on their femininity to gain advantages. For the women in Oslo as well as for the women in this study, strategies linked to sexuality were associated with the risk of victimization and marginalization. Although many women used femininity and sex in a strategy to gain advantages, some also described sex with men in the drug economy as part of a voluntary and positive sexuality. Others were strongly critical of strategies related to sex, since they believed it increased the sexualization of all women in the drug economy. It is clear that gender enactments, in this respect, are a response to gendered social hierarchies and expectations, but that they can also reproduce and reinforce them (21).

Display of street masculinity was especially important for the women who occupied relatively high or central positions in the drug economy–those who had many contacts and did business where large amounts of money or drugs were involved. There have been similar findings in other studies of drug economies (15, 33), and toughness and readiness for violence has been described as a central part of the “code of the street” and as crucial to holding high positions within criminal street environments (47). The main benefits of using street masculinity seem to be about gaining respect and reducing the risk of being robbed or out-competed (43). Disadvantages can, according to some women, be about a “split identity,” difficulties in finding a male partner to start a love relationship with, and being forced to “sacrifice close relationships” for the criminal career. For some of the women, the display of street masculinity was somewhat natural. Others said this was a “mask they were forced to put on” in certain situations or contexts. For most women, street masculinity was not an option, as they considered themselves “lacking what it takes,” felt that it is was not worth the risks, or believed there were better strategies to achieve success. A study by McNeil et al. (10) showed that not only women, but also marginal men who cannot occupy dominant roles due to age, disability, or health status are exposed to violence and subordination due to a hegemonic form of masculinity within street-based drug scenes (10).

Enacting street masculinity seems to be especially difficult to combine with motherhood. Being a mother can, according to several women, mean that you are expected to take care of the household and family. Motherhood can also entail an increased stigma and condemnation in relation to drug use and illegal activities as well as being considered as a risk or burden within criminal networks (48, 49). Grundetjern's (49) study on motherhood among women dealers in Norway showed that the women took on different maternal identities in relation to the timing off pregnancy, time spent with children, control of drug use, and place in the drug market hierarchy. Despite many challenges, some mothers described being able to successfully combine motherhood and dealing (49).

The third strategy, which includes establishing trust, being professional, and keeping a low profile, seems to reduce the risk of police interventions, conflicts and exposure to sexual harassment and abuse. Several of the women who used this strategy showed pride in working hard for their money, without having to sell sex or use violence, and compared their business to regular work or to being a “legitimate entrepreneur.” At the same time, it seems difficult to achieve higher positions in the drug economy without also being able to display street masculinity.

The fact that women in illegal economies use a business model based on relationships and service mindedness has been noted in several previous studies. Grundetjern (33) showed how women dealers in Oslo use what she called a “feminine business model,” playing on the dominant views of femininity and using care, sociability and service mindedness to gain trust. Other studies describe how successful women in the drug economy use a business ethics based on trust, fairness and efficiency, as an alternative to violence and domination (6, 11, 50). Anderson (13) even argues that men's structural sources of power in the drug market are dependent on women's roles, relational skills and power.

Although the drug economy in Malmö is generally male-dominated, there appear to be local variations and also mobility over time. Even though Malmö is a relatively small city, the drug economy seems to be made up of a number of different networks and many relatively independent actors. Several women conducted their own business independently of large criminal networks and some stated that far from all criminal men have negative attitudes toward women.

The local context and the structure and character of the drug economy have previously been shown to influence women's opportunities and roles (51). Drug economies based on small-scale or more family-oriented business models as well as on long-term relationships have been shown to work to women's advantage, resulting in a high level of female participation in market activities (20). Drug economies in Nordic countries are usually composed of small and flexible networks, with fewer large or mafia-like criminal organizations (52), which seems to be in line with the drug scene in Malmö. This could be one explanation for the fact that women in Nordic cities such as Malmö and Oslo (33) appear to have active roles and relatively good opportunities for success in the drug economy. By contrast, large, concentrated drug scenes seem to pose great risks and offer less opportunity for women, as well as for marginalized men (10).

It is clear that gender norms and gender enactments are important for the interviewed women's opportunities to take care of business. At the same time, the women's stories show that a number of other factors and circumstances also have an impact on their opportunities and risks. This points to the importance of not focusing solely on gender norms or patriarchal structures (20). For instance, factors such as social context, class, ethnicity, age and type of drug market have been shown to influence the opportunities, risks and positions of women in drug economies (51).

For the women in this study, the type of drug used, and the degree of drug dependence, was decisive for their social situation, income strategies, sexuality and risk exposure. About half of the women interviewed stated that heroin was their main drug and about half primarily used amphetamines. These are the two most commonly used drugs among people who inject drug in Sweden (2). In previous studies, heroin use has been associated with stronger dependence, higher financial costs, more illegal income strategies, sex selling, and greater social marginalization compared to amphetamine use (2, 53). These differences also appear to be true for the women in this study. More women with heroin as the main drug described problems and loss of control of their drug use and a difficult social situation. This is something that can involve difficulties in maintaining a regular job and handling illegal businesses that require planning and control, such as dealing or large-scale theft. Studies have shown that those who cannot control their drug use face major problems in pursuing a career in the drug economy (6, 11) and that the highest positions are often held by those who do not themselves use drugs to any great extent (42).

For a few women mainly using heroin, a difficult social situation combined with severe drug addiction and withdrawal symptoms resulted in short-term perspectives and temporary solutions focusing on attaining enough money for the next fix. For this group, supply strategies were reduced to shoplifting, selling sex on the street or offering sex for drugs, leading to a life situation characterized by great vulnerability and risk. Sex sales can, according to several women's stories, lead to a negative spiral where you sell sex to get money for heroin and use more heroin to deal with pain and psychological stress associated with sex sales.

It was more common among women amphetamine users to describe a relatively stable social situation and a greater opportunity to choose between different sources of income. Amphetamine was also used by some of the women to help them endure long and hard working days. Fewer women using amphetamine regularly sold sex. At the same time, it was more common for women who mainly used amphetamine to have temporary sexual contacts with men in the drug world, both as a strategy to gain advantages, but also as part of a positive sexuality. The differences in sexuality between women using heroin and women using amphetamine can partly be explained by the fact that amphetamine is a sexual drug that can eliminate sexual inhibition, increase sexual desire, pleasure and stamina (54, 55), whereas heroin and other opioids rarely are associated with a positive sexuality (56).

It is clear from the woman's stories that the type of drug used, the different functions of the drug, as well as the degree of drug dependence play an important role for their everyday life and risks as well as for their positions and success within the drug economy. This is something that has received relatively little attention in research, and which therefore needs to be explored in more depth in future research.

The women's stories show how they are restrained by gender norms and structures, how they use them to their advantage and how in some cases they also contribute to maintaining and reproduce them. Their stories also provide examples of resistance to sexism and to stereotypical and negative perceptions of female drug users, and how they use and move between multiple femininities or masculinity to achieve success.

Some of the stories stand out and provide more alternative images of the drug economy and of women who use drugs. They show, for example, that gender is not always decisive to women's opportunities and that far from all men in the drug economy hold negative or prejudiced views of women, that it is possible as a woman to be successful and reach high positions in a male-dominated and criminal context, that multiple sexual relationships under the influence of drugs do not necessarily have to be destructive or harmful, the possibility to combine a family- and working life with regular injection drug use, and that it is possible to engage in criminal activities such as drug dealing based on ethical codes and a sense of pride.

## Conclusions

It is possible for women to establish themselves as successful entrepreneurs in male-dominated drug economies, but this is associated with major obstacles and risks that are highly gendered. Displaying toughness and street masculinity has been presented as crucial for success in street level drug economies. The women's stories indicate that this is only one of many possible strategies for achieving respect and success. Establishing trust, being professional, and keeping a low profile, can in some contexts and situations, be a better strategy than “being cocky” or using violence. Using sex or playing on sexuality can open opportunities for women in male-dominated contexts, but this can also involve increased risks of sexual abuse and a lower social status.

Similar to a number of previous studies, the results show that gendered social hierarchies, structures and norms are of great importance for women's gender enactments, opportunities and risks in drug economies. At the same time, the results point to the importance of also focusing on other factors. The person's drug of choice, degree of drug dependence and social situation can in many cases be of great significance for risks and the possibility of taking care of business. The results also indicate that drug economies and gender norms can rarely be understood as static or uniform. Drug economies based on small scale business models seem to be more flexible and heterogeneous, meaning they offer greater opportunities for women to succeed in business.

## Limitations

The study is based on interviews with a highly selected group of women–women with long-term injection use of heroin or amphetamine–within a specific context. This means that the results cannot be generalized to women's roles, opportunities or vulnerabilities in drug economies in general. However, there are a number of themes in the results that show great similarities with those presented in several previous studies. This indicates the possibility of a certain thematic or theoretical generalizability. Nevertheless, the results must always be understood based on the specific features of the local context.

The group of women included in the study is in some respects homogeneous. The women were all white, and all but two were born in Sweden. This means that questions relating to having an ethnic minority position, and how this may affect women's experiences and opportunities have not been made visible. All interviewees were cisgender women, and none of the women talked about homo- or bisexual relationships or sexual experiences. This is one explanation for the relatively dichotomous and heteronormative views on sexuality and gender roles in the women's narratives, and for the lack of a queer or transgender perspective in the study. A main focus in the interviews and analysis was on gender hierarchies and gender enactments. Although a certain focus was also directed toward the type of drug used, the degree of dependence as well as the women's social situation, other important aspects of their everyday lives may have been overshadowed.

## Data Availability Statement

The original contributions presented in the study are included in the article/supplementary material, further inquiries can be directed to the corresponding author.

## Ethics Statement

The studies involving human participants were reviewed and approved by the Regional Ethical Review Board at Lund University (ref.nr. 2006-346). The patients/participants provided their written informed consent to participate in this study.

## Author Contributions

TR is the sole author of the article, conducted all interviews, transcribed them and conducted the analyses, and also wrote the first draft, as well as the final manuscript.

## Conflict of Interest

The author declares that the research was conducted in the absence of any commercial or financial relationships that could be construed as a potential conflict of interest.

## Publisher's Note

All claims expressed in this article are solely those of the authors and do not necessarily represent those of their affiliated organizations, or those of the publisher, the editors and the reviewers. Any product that may be evaluated in this article, or claim that may be made by its manufacturer, is not guaranteed or endorsed by the publisher.
